# Repeated leftover serosurvey of SARS-CoV-2 IgG antibodies, Greece, March and April 2020

**DOI:** 10.2807/1560-7917.ES.2020.25.31.2001369

**Published:** 2020-08-06

**Authors:** Zacharoula Bogogiannidou, Alexandros Vontas, Katerina Dadouli, Maria A Kyritsi, Soteris Soteriades, Dimitrios J Nikoulis, Varvara Α Mouchtouri, Michalis Koureas, Evangelos I Kazakos, Emmanouil G Spanos, Georgia Gioula, Evangelia E Ntzani, Alexandros A Eleftheriou, Alkiviadis Vatopoulos, Efthimia Petinaki, Vassiliki Papaevangelou, Matthaios Speletas, Sotirios Tsiodras, Christos Hadjichristodoulou

**Affiliations:** 1 Laboratory of Hygiene and Epidemiology, Faculty of Medicine, University of Thessaly, Larissa, Greece; 2 Faculty of Midwifery, School of Health Sciences, University of Western Macedonia, Kozani, Greece; 3 National Influenza Reference Laboratory for Northern Greece, Microbiology Department, Medical School, Aristotle University of Thessaloniki, Thessaloniki, Greece; 4 Department of Hygiene and Epidemiology, University of Ioannina Faculty of Medicine, Ioannina, Greece; 5 Center for Evidence Synthesis in Health, Department of Health Services, Policy and Practice, School of Public Health, Brown University, Providence, United States; 6 Institute of Biosciences, University Research Center of loannina, Ioannina, Greece; 7 Department of Public Health Policy, School of Public Health, University of West Attica, Athens, Greece; 8 Department of Microbiology, University Hospital of Larissa, University of Thessaly, Larissa, Greece; 9 Third Department of Paediatrics, National and Kapodistrian University of Athens, School of Medicine, Attikon University Hospital, Athens, Greece; 10 Department of Immunology and Histocompatibility, Faculty of Medicine, University of Thessaly, Larissa, Greece; 11 Fourth Department of Internal Medicine, National and Kapodistrian University of Athens, School of Medicine, Attikon University Hospital, Athens, Greece

**Keywords:** COVID-19, serosurvey, SARS-CoV-2 IgG antibodies, Greece

## Abstract

A serosurvey of IgG antibodies against severe acute respiratory coronavirus 2 (SARS-CoV-2) was performed during March and April 2020. Among 6,586 leftover sera, 24 (0.36%) were positive, with higher prevalence in females, older individuals and residents of large urban areas. Seroprevalence was estimated at 0.02% and 0.25%, respectively, in March and April, infection fatality rate at 2.66% and 0.54%. Our findings confirm low COVID-19 incidence in Greece and possibly the effectiveness of early measures.

On 10 March 2020, with 89 active coronavirus disease (COVID-19) cases and 0 deaths reported, the Greek government decided to suspend the operation of all educational institutions throughout the country [1]. Gradually, restrictive measures were extended and resulted in a general lockdown on 23 March [2], when 649 active cases and two deaths had been recorded. By 30 April 2020, Greece had reported 2,310 laboratory confirmed cases of severe acute respiratory syndrome coronavirus 2 (SARS-CoV-2) infection in the general population and 140 related deaths [3]. The recorded cumulative incidence of COVID-19 in Greece until 30 April was estimated at 24.3 cases per 100,000 population and the mortality at 1.3 deaths per 100,000 population, which are considered low in comparison with other countries worldwide [4]. 

The aims of the present sero-epidemiological study were to estimate the prevalence of severe acute respiratory coronavirus 2 (SARS-CoV-2) IgG antibodies in the population of Greece by sex, age group and geographical area, to provide evidence for the potential underdiagnosis of COVID-19 in Greece, to identify regional differences in order to improve surveillance and finally, to assess the infection fatality rate (IFR) and compare it to the case fatality rate (CFR).

## Study design and participants

Blood samples were collected by using the leftover sampling methodology (residual sera from the general population) [5]. We applied a geographically stratified sampling plan based on regional units (NUTS level 3) to produce a representative sample, taking into consideration age group (0–29, 30–49, 50–69, ≥ 70 years) and sex. The required sample size was determined to be 380 blood samples from each of the 13 NUTS level 2 regions, and the sample size for each regional unit (NUTS level 3) from the corresponding region was calculated according to population distribution. However, the number of actual samples collected differed from the pre-determined number of samples above. The study was designed as a cross-sectional survey and repeated at monthly intervals. Here we present the results from March and April 2020. The leftover blood samples were collected from a nationwide laboratory network, including both private and public hospital laboratories (27 laboratories in total). The samples were collected from individuals who visited the laboratories for a check-up, chronic disease follow-up or other reasons unrelated to COVID-19. The geographical distribution of the collected leftover samples is shown in the Figure. We collected 1,575 samples from Central Macedonia, 1,133 from Attica, 1,115 from Peloponnese, 853 from Thessaly, 759 from Western Macedonia, 494 from Central Greece, 371 from Western Greece and 286 from Epirus. Age, sex, residence and the date of blood sampling were recorded.

**Figure:**
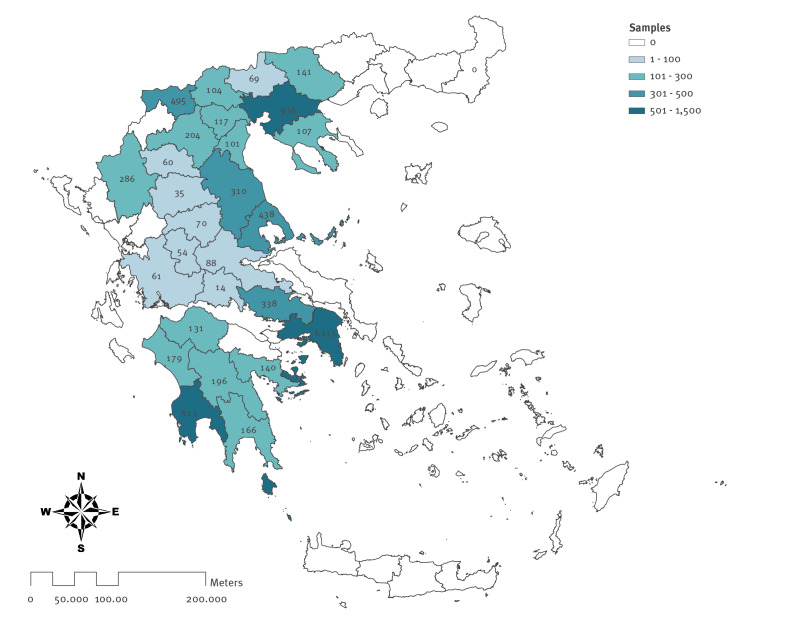
Geographical distribution of leftover samples collected for COVID-19 serosurvey, Greece, March–April 2020 (n = 6,586)

## Laboratory analysis

The presence of IgG antibodies against SARS-CoV-2 was determined using the ABBOTT SARS-CoV-2 IgG assay, a chemiluminescent microparticle immunoassay (CMIA), with the ARCHITECT i2000SR analyzer (Abbott, Illinois, United States). The method was validated in our laboratory using 305 pre-COVID-19 samples (obtained in 2017) as negative controls and 94 samples from patients with positive SARS-CoV-2 PCR and different symptom durations. The kit displayed 84.0% sensitivity (95% confidence interval (CI): 76.6–91.5) and 99.7% specificity (95% CI: 98.2–100). All positive samples, as well as 100 randomly chosen samples negative in the ABBOTT assay were confirmed with ELISA kits such as the Vircell COVID-19 IgG (Vircell Spain S.L.U., Granada, Spain) and the Euroimmun SARS-CoV-2 IgG (Euroimmun, Lübeck, Germany).

## Statistical analysis

### Weighted prevalence

Initially, we determined an unweighted relative frequency of all patient characteristics (age, sex and area of residence): this is the crude seroprevalence (S1). The weighted proportions of positive tests in the countrywide sample were based on the sex and age distribution within each regional unit (NUTS level 3) and the population of each regional unit, according to the 2011 census (S2) [6]. We also adjusted the weighted proportion (S2) of positive tests to account for the accuracy (sensitivity and specificity) of the laboratory test (S3) [7-9]. Since the reported COVID-19 cases were by definition outside the sampling framework, the seroprevalence was corrected, taking into consideration the number of reported cases per month in accordance with the National Public Health Organisation (NPHO) (S4).Therefore, we added the cases reported in March to the estimated S3 seroprevalence in order to calculate the S4 for March, while to calculate the S4 for April, we added the reported cases from March and April. We calculated the S1, S2, S3 and S4 seroprevalence of IgG antibodies by month, the CFR and the IFR. CFR is the ratio of the number of deaths attributed to COVID-19 and reported to the NPHO, divided by the number of cases reported to the NPHO. IFR is the ratio of deaths divided by the number of estimated people infected with SARS-CoV-2. The estimation of infected people was the product of the seroprevalence and the population of regional units where confirmed cases were found according to NPHO [3,10,11]. The 95% CI for weighted data were estimated using normal approximation of binomial distribution and effective sample size instead of the collected sample size, further explained below. It should be noted that clusters of cases from refugee camps and from a cruise ferry that were not considered community cases (302 cases in total) were excluded from the analysis for CFR and IFR. The 95% CI for CFR were calculated using normal approximation of binomial distribution. The 95% CI for IFR were calculated using the corresponding 95% CI of the S1, S2, S3 and S4 seroprevalence. Comparison of two proportions was done with the 'N-1' chi-squared test [10]. For all analyses, a 5% significance level was set.

### Effective sample size

Since the number of collected samples from each regional unit was not proportional to the regional unit’s population, we calculated an effective sample size based on each regional unit’s population proportion, according to 2011 census data. This was done using target weighting. The target sample size for a regional unit *i* is *t_i_*, and the actual sample size for the regional unit *i* is *a_i_*. The weighting factor for the regional unit *i* is calculated with the following formula:

$$f_{i}=\frac{t_{i}}{a_{i}}\;\;\;\;\;\;\;\;\;\;\;\;\;\;\;\;\;\;(Formula\;1)$$

The weighted sample size (*w_i_*) for the regional unit *i* is calculated as follows:

wi = ti × fi                  (Formula 2)

For *k* regional units and a countrywide target sample size of *n_t_*, the country-wide effective sample size (*n_e_*) is calculated with the following formula:

$$n_{e}=\frac{{n_{t}}^{2}}{\sum_{i=1}^{k}w_{i}}\;\;\;\;\;\;\;\;\;\;(Formula\;3)$$

This can also be written as:

$$n_{e}=\frac{({\sum_{i=1}^{k}t_{i})}^{2}}{\sum_{i=1}^{k}\frac{{t_{i}}^{2}}{a_{i}}}\;\;\;\;\;\;\;\;\;\;\;\;(Formula\;4)$$

### Ethical statement

The data were anonymised and the laboratories requested a written consent statement from the participants. The study was approved by the ethical committee of the Faculty of Medicine, University of Thessaly (No. 2116).

## Serosurvey results

Twenty-four of 6,586 (0.36%) collected samples were found positive for anti-SARS-CoV-2 IgG antibodies. Regarding samples from March, five of 2,075 (S1 = 0.24%) were positive (Table 1), while 19 of 4,511 (S1 = 0.42%) samples from April were IgG positive (Table 2). As shown in Tables 1 and 2, the S2 and S3 seroprevalences were higher in April than in March: S2 = 0.49% and S3 = 0.23% in April vs S2 = 0.27% and S3 = 0% in March. The S2 and S3 among females were higher than among males for both months, with the higher percentage occurring in April (females: S2 = 0.94%, S3 = 0.76% vs males: S2 = 0.46%, S3 = 0.19%). Moreover, in large urban areas (Attica region and Thessaloniki regional unit) in April, the S2 (0.99%) and S3 (0.83%) were higher than the estimated S2 (0.27%) and S3 (0%) in the rest of the country. A gradual increase of S4 by age was prominent in April, from 0.02% in the age group 0–29 years to 1.17% in the age group ≥ 70 years.

**Table 1:** Anti-SARS-CoV-2 IgG antibody seroprevalence, Greece, March 2020 (n = 2,075)

March		Positive/ sample size	S1: Crude prevalence		S2: Age, sex and population-adjusted prevalence		S3: S2 + adjustment for sensitivity and specificity		S4: S3 + NPHO data^a^	
		n/N	Prevalence (%)	95% CI	Prevalence (%)	95% CI	Prevalence (%)	95% CI	Prevalence (%)	95% CI
**Total**		**5/2,075**	**0.24**	**0.03–0.45**	**0.27**	**0.05–0.49**	**0**	**0–0.23**	**0.02**	**0–0.25**
Age group (years)	0–29	0/490	0		0		0		0.01	0–0.09
	30–49	0/695	0		0		0		0.01	0–0.10
	50–69	3/533	0.56	0–1.20	0.75	0.02–1.48	0.54	0–1.41	0.56	0.02–1.43
	≥ 70	2/357	0.56	0–1.33	0.55	0–1.31	0.29	0–1.21	0.30	0.01–1.22
Sex	Male	1/928	0.11	0–0.32	0.15	0–0.39	0	0–0.11	0.01	0–0.12
	Female	4/1,147	0.35	0.01–0.69	0.40	0.03–0.76	0.12	0–0.55	0.14	0–0.32
‘Ν-1’ chi-squared test Difference between sex			Difference = 0.24% p = 0.269		Difference = 0.25% p = 0.291		Difference = 0.12% p = 0.291		Difference = 0.13% p = 0.303	
Large urban areas		4/1,072	0.37	0.01–0.74	0.35	0–0.71	0	0–0.37	0.02	0–0.36
Rest of country		1/1,003	0.10	0–0.3	0.13	0–0.35	0	0–0.05	0.01	0–0.06
‘Ν-1’ chi-squared test Difference between large urban areas and rest of country			Difference = 0.27% p = 0.209		Difference = 0.22% p = 0.310		NA		Difference = 0.01% p = 0.853	
CFR (%)		95% CI	IFR according to							
			**S1**		**S2**		**S3**		**S4**	
			IFR (%)	95% CI	IFR (%)	95% CI	IFR (%)	95% CI	IFR (%)	95% CI
3.61		2.63–4.59	0.22	0.12–1.77	0.20	0.11–1.14	NA		2.66	0.64-NA

CFR: case fatality rate; CI: confidence interval; IFR: infection fatality rate; NA: not applicable; NPHO: National Public Health Organisation.

^a^ NPHO data include all confirmed PCR-positive individuals.

**Table 2:** Anti-SARS-CoV-2 IgG antibody seroprevalence, Greece, April 2020 (n = 4,511)

April		Positive/ sample size	S1: Crude prevalence		S2: Age, sex and population-adjusted prevalence		S3: S2 + adjustment for sensitivity and specificity		S4: S3 + NPHO data^a^	
		n/N	Prevalence (%)	95% CI	Prevalence (%)	95% CI	Prevalence (%)	95% CI	Prevalence (%)	95% CI
**Total**		**19/4,511**	**0.42**	**0.23–0.61**	**0.49**	**0.29–0.70**	**0.23**	**0–0.48**	**0.25**	**0.02–0.50**
Age group (years)	0–29^b^	4/974	0.41	0.01–0.81	0.23	0–0.54	0	0–0.28	0.02	0.02–0.29
	30–49	2/1371	0.15	0–0.35	0.09	0–0.25	0	0–0.01	0.03	0.02–0.04
	50–69	5/1229	0.41	0.05–0.76	0.86	0.34–1.38	0.67	0.05–1.29	0.70	0.09–1.32
	≥ 70	8/937	0.85	0.26–1.44	1.26	0.55–1.98	1.15	0.30–2.00	1.17	0.32–2.02
Sex	Male	6/2,073	0.29	0.06–0.53	0.46	0.17–0.76	0.19	0–0.54	0.22	0.03–0.57
	Female	13/2,474	0.53	0.24–0.81	0.94	0.56–1.32	0.76	0.31–1.21	0.78	0.33–1.23
‘Ν-1’ chi-squared test Difference between sex			Difference = 0.24% p = 0.213		Difference = 0.48% p = 0.057		Difference = 0.57% p = 0.007		Difference = 0.56% p = 0.009	
Large urban areas		9/997	0.90	0.32–1.49	0.99	0.38–1.61	0.83	0.09–1.56	0.85	0.11–1.58
Rest of country		10/3,514	0.28	0.11–0.46	0.27	0.10–0.45	0	0–0.18	0.01	0–0.19
‘Ν-1’ chi-squared test Difference between large urban areas and rest of country			Difference = 0.62% p = 0.007		Difference = 0.72% p = 0.021		Difference = 0.83% p < 0.001		Difference = 0.83% p < 0.001	
CFR (%)		95% CI	IFR according to:							
			**S1**		**S2**		**S3**		**S4**	
			IFR (%)	95% CI	IFR (%)	95% CI	IFR (%)	95% CI	IFR (%)	95% CI
6.06		5.09–7.03	0.33	0.22–0.59	0.28	0.20–0.47	0.59	0.29–NA	0.54	0.27–6.85

CFR: case fatality rate; CI: confidence interval; IFR: infection fatality rate; NA: not applicable; NPHO: National Public Health Organisation.

^a^ NPHO data include all confirmed PCR-positive individuals.

^b^ A 12-year-old girl was detected positive for SARS-COV-2 IgG antibodies among 182 children aged 0–14; thus, the S4 is estimated to be 0.001% (95% CI: 0–0.09%) for the age group 0–14 years.

By using the total reported cases (2,310) until 30 April 2020 and the S3 seroprevalence, we estimated that every case confirmed by RT-PCR corresponded to 10.2 (95% CI: 0–21.2) COVID-19 cases in the Greek population.

Finally, according to NPHO data and our results, the CFR in March was calculated as 3.61% and the IFR as 2.66%; for April the corresponding values were 6.06% and 0.54% for CFR and IFR, respectively (Tables 1 and 2).

## Discussion

The reported incidence and mortality of COVID-19 during March and April 2020 in Greece were among the lowest in Europe. During the same period, 75,170 RT-PCR tests (which corresponds to a rolling 7-day average of 0.22 per 1,000 population for 23–30 April) were conducted, mainly among persons with moderate to severe COVID-19-compatible symptoms [3,11]. As the rate of testing was rather low, there were concerns regarding both the actual number of cases recorded in Greece, and the potential overestimation of the CFR of the disease [12].

Our study demonstrates low seroprevalence of SARS-CoV-2 in Greece (0.02% for March and 0.25% for April) during the first 2 months of the COVID-19 pandemic. The low seroprevalance in the Greek population may further support the hypothesis that the early implementation of public health measures in Greece resulted in the low incidence and mortality. Nevertheless, the 10-fold higher percentage in April demonstrates that community circulation of the virus had increased during that period.

A second finding of note was the higher seroprevalence in females (0.78% vs 0.22% in males). This finding contradicts reports from the NPHO in which 56% of cases until 30 April 2020 were male [3]. This difference could be attributed to convenient and non-random sampling methodology. However, at the beginning of the pandemic, only moderately or severely affected individuals were tested for SARS-CoV-2 infection in accordance with the national testing strategy. Male patients are more vulnerable to experiencing complications (73.6% of deaths and 73.7% of intensive care unit hospitalisations in Greece were males according to NPHO [3]) and as such were more likely to be tested for SARS-CoV-2. Seroprevalence is a measure of exposure and does not necessarily correlate with the severity of symptoms experienced. It should be noted that no difference was found in seroprevalence between females and males in other serosurveys conducted in Europe [13,14]. However, since we continue the serosurvey (samples for May and June have been collected and are being analysed), we have the opportunity over the next few months to further explore if this finding is consistent.

We noted a higher seroprevalence of COVID-19 in individuals 70 years and older (1.17%), suggesting that this age group was most exposed. In accordance with the characteristic age distribution for COVID-19 cases [15], we observed higher seroprevalence with increasing age. Moreover, higher seroprevalence (0.85%) was also found in large urban areas when compared with the rest of the country (0.01%), which could be explained by larger populations, crowded conditions as well as by earlier importation of COVID-19 cases from abroad. The above findings are in concordance with a recently published Spanish seroprevalence study [13].

Furthermore, the finding that for every laboratory-confirmed COVID-19 case there were ca additional 10 cases, is in accordance with a recent serosurvey conducted in Switzerland [14]. This indicates the necessity of increasing testing capacity in Greece, which could potentially allow diagnosing mild or asymptomatic cases [16]. The same ratio of ca 10 was identified when we compared the estimated CFR (6.06%) with the IFR (0.59%) in April 2020. It should be noted that both CFR and IFR are estimated within a specific timeframe and the delay of death could not be accounted for in the current estimation.

The leftover sampling methodology could be considered a limitation of our study; mainly the non-random convenient sampling may affect the representativeness of the collected samples. Moreover, certain areas were not covered by the sampling framework although in total, the samples were collected from areas representing almost 90% of the Greek population. Owing to the general lockdown that was implemented, the number of routine laboratory tests was reduced and it was challenging to collect the estimated number of samples. However, this methodology has the advantage of easy sample collection and the option of repeating collection on a monthly basis, which allows following up the epidemic and drawing conclusions about public health strategies.

## Conclusion

Our study demonstrates a low seroprevalence for COVID-19 in Greece in accordance with relatively low incidence, compared with other European Union countries. Although these findings could be attributed to the early implementation of public health measures, further research is needed to elucidate this issue. The low seroprevalence render the Greek population particularly vulnerable to a possible second COVID-19 wave and should be taken into consideration when drafting the future plan of action.
